# Characterization of hemin-binding protein 35 (HBP35) in *Porphyromonas gingivalis*: its cellular distribution, thioredoxin activity and role in heme utilization

**DOI:** 10.1186/1471-2180-10-152

**Published:** 2010-05-25

**Authors:** Mikio Shoji, Yasuko Shibata, Teruaki Shiroza, Hideharu Yukitake, Benjamin Peng, Yu-Yen Chen, Keiko Sato, Mariko Naito, Yoshimitsu Abiko, Eric C Reynolds, Koji Nakayama

**Affiliations:** 1 Division of Microbiology and Oral Infection, Department of Molecular Microbiology and Immunology, Nagasaki University Graduate School of Biomedical Sciences, Nagasaki, Japan; 2 Department of Biochemistry and Molecular Biology, Nihon University School of Dentistry at Matsudo, Chiba, Japan; 3 Cooperative Research Centre for Oral Health Science, Melbourne Dental School, University of Melbourne, Victoria, Australia; 4 Global COE Program at Nagasaki University, Nagasaki, Japan

## Abstract

**Background:**

The periodontal pathogen *Porphyromonas gingivalis *is an obligate anaerobe that requires heme for growth. To understand its heme acquisition mechanism, we focused on a hemin-binding protein (HBP35 protein), possessing one thioredoxin-like motif and a conserved C-terminal domain, which are proposed to be involved in redox regulation and cell surface attachment, respectively.

**Results:**

We observed that the *hbp35 *gene was transcribed as a 1.1-kb mRNA with subsequent translation resulting in three proteins with molecular masses of 40, 29 and 27 kDa in the cytoplasm, and one modified form of the 40-kDa protein on the cell surface. A recombinant 40-kDa HBP35 exhibited thioredoxin activity *in vitro *and mutation of the two putative active site cysteine residues abolished this activity. Both recombinant 40- and 27-kDa proteins had the ability to bind hemin, and growth of an *hbp35 *deletion mutant was substantially retarded under hemin-depleted conditions compared with growth of the wild type under the same conditions.

**Conclusion:**

*P. gingivalis *HBP35 exhibits thioredoxin and hemin-binding activities and is essential for growth in hemin-depleted conditions suggesting that the protein plays a significant role in hemin acquisition.

## Background

*Porphyromonas gingivalis *has been implicated as a major pathogen associated with chronic periodontitis. The establishment of *P. gingivalis *at a periodontal site and progression of disease is dependent on the ability of the bacterium to utilize essential nutrients, of which iron (preferably in the form of heme) plays a crucial role. *P. gingivalis *lacks the majority of enzymes in the biosynthetic pathway for the porphyrin ring, hence it is unable to synthesize protoporphyrin IX, the precursor of heme [1-3]; and unlike other Gram-negative bacteria, *P. gingivalis *does not produce siderophores [3]. Although several studies have shown that *P. gingivalis *acquires heme from the host environment using gingipains, lipoproteins and specific outer-membrane receptors [3-5], the precise mechanisms by which *P. gingivalis *acquires heme are still unknown.

The gene encoding the *P. gingivalis *outer membrane 40-kDa protein (OMP40) was first cloned by Abiko *et al*. [6]. As the recombinant OMP40 protein was demonstrated to exhibit hemin binding ability, and the molecular mass of the mature polypeptide determined by mass spectrometric analysis was 35.3 kDa, the protein was designated as HBP35 [7]. However, characterization of the *hbp35 *gene at the transcriptional and translational levels in *P. gingivalis *and contribution of HBP35 protein to hemin utilization have not been elucidated.

HBP35 protein has unique characteristics including the presence of one thioredoxin-like motif and a conserved C-terminal domain. Recently, it has been reported that the C-terminal domain of a group of *P. gingivalis *outer membrane proteins plays a crucial role in the coordinated process of exportation and attachment of those proteins onto the cell surface [8] and that some of the C-terminal domain containing proteins, including RgpB, are glycosylated [9,10]. The last five residues of the C-terminal domain are well conserved not only in *P. gingivalis *but also in other oral pathogens, and that the last two C-terminal residues (VK) of RgpB have been shown to be essential for correct transport and posttranslational modification [11]. However, the transportation and posttranslational modification mechanisms of C-terminal domain containing proteins other than RgpB remain poorly understood.

In this study, we presented the first evidence that the *hbp35 *gene produces three translational products in *P. gingivalis*. One was a 40-kDa protein that was transported to the outer membrane and glycosylated on the cell surface, resulting in diffuse proteins with molecular masses of 50-90 kDa. The others were smaller truncated 29- and 27-kDa proteins. We constructed HBP35-deficient mutants to elucidate the role of the gene products in this microorganism and found that the HBP35 protein (40-kDa) exhibited thioredoxin activity and bound hemin and that its C-terminal domain was involved in its transport to the outer membrane. The protein was also essential for growth of the bacterium in a hemin-depleted environment.

## Results

### Immunoblot analysis of P. gingivalis hbp35 mutants with anti-HBP35 antibody

**Figure 1:**
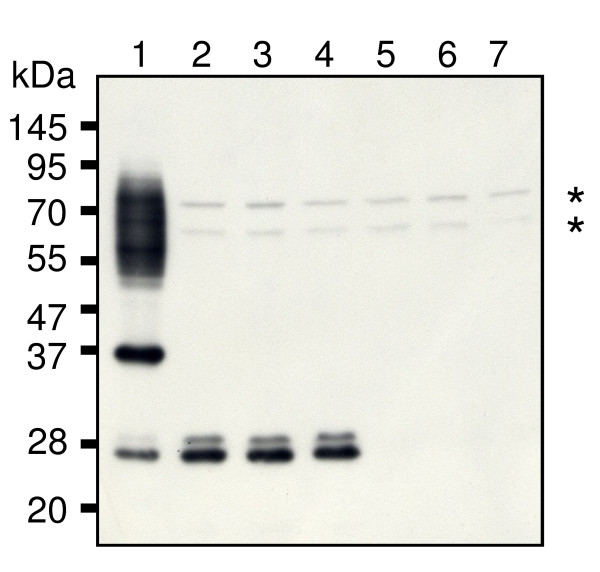
**Immunoblot analysis of cell extracts of various *P. gingivalis *strains with anti-HBP35**. Cell extracts (approximately 10 μg protein) of various *P. gingivalis *strains were analyzed by SDS-PAGE under reducing conditions followed by immunoblotting with anti-HBP35 antibody. Lane 1, 33277 (wild type); lanes 2, 3 and 4, KDP164 (*hbp35 *insertion mutant); lanes 5, 6 and 7, KDP166 (*hbp35 *deletion mutant). Asterisks indicate protein bands with molecular masses of 70-and 65-kDa non-specifically recognized by anti-HBP35 antibody.

To gain insights into the biological significance of HBP35 in *P. gingivalis*, HBP35-deficient mutants, which had full length deletion of, or insertion in, the *hbp35 *gene, were constructed from the wild-type strain 33277. Immunoblot analysis with an anti-HBP35 antibody revealed that whole cell extracts of *P. gingivalis *33277 showed three protein bands with apparent molecular masses of 40, 29, and 27 kDa and diffuse bands of 50-90 kDa (Figure 1). No protein bands other than those of 70 and 65 kDa indicated by asterisks, which might be non-specific, were detected in the *hbp35 *full length deletion mutant (KDP166), whereas the *hbp35 *insertion mutant (KDP164), which had an insertion of the *ermF-ermAM *DNA cassette just upstream of the F^110 ^residue within the HBP35 protein, showed 29-and 27-kDa proteins (Figure 1). We checked independent 18 isolates of KDP164 and 5 isolates of KDP166. All of the isolates showed the same results as shown in Figure 1. The 40-kDa protein appeared as the full length gene product of *hbp35*, which coincided with results of previous studies [6,7].

### Pigmentation and gingipain activities of P. gingivalis hbp35 mutants

Both full length deletion and insertion *P. gingivalis hbp35 *mutants formed black pigmented colonies on blood agar plates. No difference was observed in Rgp, Kgp and hemagglutinating activities between the *hbp35 *mutants and the wild type (data not shown). These results suggest that HBP35 does not influence expression of gingipain-encoding genes.

### Northern blot analysis of hbp35

To determine whether the *hbp35 *gene produces multiple transcripts, total RNAs were prepared from the wild type and *hbp35 *mutants. Northern blot analysis was then carried out with an *hbp35 *DNA probe that hybridized to the *hbp35 *region coding for Q^22^-P^344^. The wild type showed a 1.1-kb transcript hybridizing to the *hbp35 *probe (Additional file 1). In the *hbp35 *insertion and full length deletion mutants, there was no 1.1-kb transcript, indicating that the 1.1-kb mRNA was produced from the *hbp35 *gene. The *hbp35 *insertion mutant produced transcripts with 1.3-2.2 kb that hybridized to the probe. The *ermF *probe hybridized to transcripts with similar length in the *hbp35 *insertion mutant (Additional file 1).

### Subcellular localization of HBP35 protein

**Figure 2:**
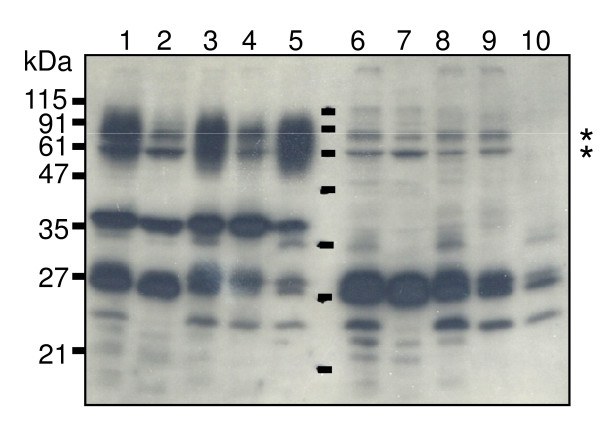
**Subcellular localization of HBP35**. Subcellular fractions of *P. gingivalis *33277 (lanes 1 to 5) and KDP164 (*hbp35 *insertion mutant) (lanes 6 to 10) were subjected to immunoblot analysis using anti-HBP35 antibody. Lanes 1 and 6, whole cells; lanes 2 and 7, cytoplasm/periplasm fraction; lanes 3 and 8, total membrane fraction; lanes 4 and 9, inner membrane fraction; lanes 5 and 10, outer membrane fraction. Horizontal lines between lane 5 and 6 indicate the molecular size marker proteins corresponding to the far left markers. Asterisks indicate the non-specific protein bands recognized by anti-HBP35 antibody.

In an approach to understand the potential roles of HBP35 proteins with different molecular masses, we fractionated cells of the wild type and the *hbp35 *insertion mutant into cytoplasm/periplasm, total membrane, and inner and outer membrane fractions. These fractions were subjected to SDS-PAGE and immunoblot analysis with the anti-HBP35 antibody. The diffuse bands of 50-90 kDa proteins were mainly detected in the outer membrane fraction, whereas the 40-kDa protein was detected in every fraction of the wild type but mainly in the inner membrane fraction. The 29-and 27-kDa proteins were mainly detected in the cytoplasm/periplasm fraction of the wild type and *hbp35 *insertion mutant (Figure 2).

### Peptide Mass Fingerprint analysis of the 27-kDa protein

**Figure 3:**
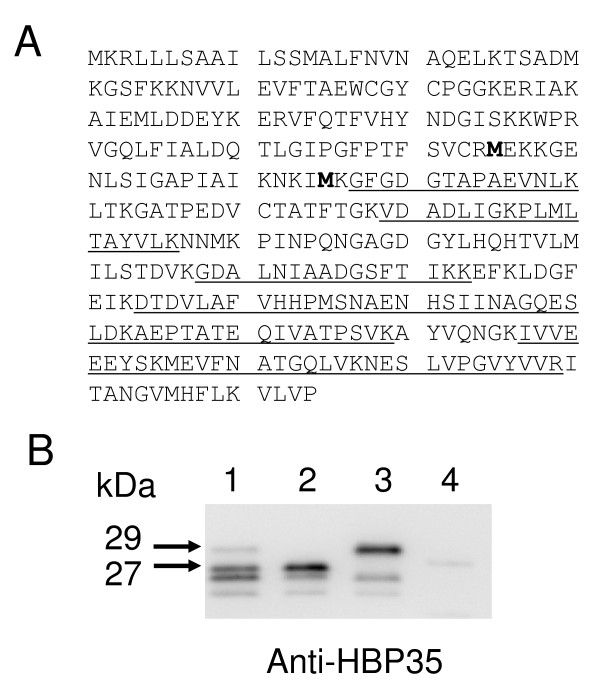
**Identification of the anti-HBP35-immunoreactive 27-kDa protein and the start codons of anti-HBP35-immunoreactive proteins**. A. PMF analysis of the anti-HBP35-immunoreactive 27-kDa protein from KDP164 (*hbp35 *insertion mutant). Underlined peptide fragments were indicated by the PMF data of the protein. Bold letters indicating M^115 ^and M^135 ^were suspected to be internal start codons. B. Immunoblot analysis of *P. gingivalis *mutants with various amino acid substitutions of HBP35 protein. Lane 1, KDP164 (*hbp35 *insertion mutant); lane 2, KDP168 (*hbp35 *[M^115^A] insertion mutant); lane 3, KDP169 (*hbp35 *[M^135^A] insertion mutant); lane 4, KDP170 (*hbp35 *[M^115^A M^135^A] insertion mutant).

To determine whether the 27-kDa protein is a truncated form of the HBP35 protein, an immunoprecipitation experiment using the *hbp35 *insertion mutant (KDP164) cell lysate was carried out with the anti-HBP35 antibody. The resulting immunoprecipitate contained a 27-kDa protein band (Additional file 2), which was digested with trypsin followed by MALDI-TOF mass spectrometric analysis. The 27-kDa protein was found to be derived from a 3'-portion of *hbp35*, with PMF sequence coverage of 37% of full length protein (Figure 3A). The maximum mass error among the identified 10 tryptic peptides was 14 ppm. Since the detected tryptic peptide located at the most N-terminal region of HBP35 starts from G^137 ^and since the insertion site of the *ermF-ermAM *DNA cassette in the insertion mutant is just upstream of F^110^, it is feasible that the 27-kDa protein uses M^115 ^or M^135 ^as the alternative translation initiation site.

### Identification of the N-terminal amino acid residue of truncated HBP35 proteins

To clarify the N-terminal amino acid residue of the truncated HBP35 proteins, we introduced amino acid substitution mutations of [M^115^A] or/and [M^135^A] to the *hbp35 *insertion mutant (KDP164) producing the 29-and 27-kDa HBP35 proteins (Additional file 3). As shown in Figure 3B, the 27-kDa protein was not observed in the *hbp35 *[M^135^A] insertion mutant (KDP169), while the 29-kDa protein was not observed in the *hbp35 *[M^115^A] insertion mutant (KDP168), suggesting that M^115 ^and M^135 ^are the N-terminal amino acid residues of the 29-and 27-kDa proteins, respectively. The use of M^115 ^and M^135 ^as alternative translation initiation sites was supported by the finding that no HBP35 translational product was detected in the *hbp35 *[M^115^A and M^135^A] insertion mutant (KDP170). Moreover, recombinant HBP35 proteins with a C-terminal histidine-tag were produced in an *E. coli *strain expressing the *hbp35 *gene and purified by a histidine-tag purification system. Immunoblot analysis revealed that the purified products contained 40-, 29-, and 27-kDa proteins immunoreactive to the anti-HBP35 anitibody. Edman sequencing revealed that the N-terminal amino acid residue of the recombinant 27-kDa protein was M^135 ^(Additional file 4).

### Hemin binding site of rHBP35 proteins

**Figure 4:**
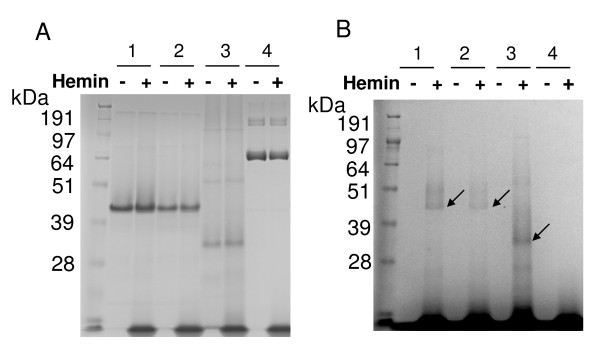
**Hemin binding of various rHBP35 proteins**. Two μg each of rHBP35(Q^22^-P^344^) (lane 1), rHBP35 (Q^22^-P^344 ^with C^48^S C^51^S) (lane 2), truncated rHBP35(M^135^-P^344^) (lane 3), or lactoferrin as a negative control (lane 4) was treated with or without 1.5 μl of 1.25 mM hemin for 2 h at room temperature. A, CBB staining; B, peroxidase activity staining. Arrowheads indicate the hemin binding proteins.

Shibata *et al*. [7] found that a purified rHBP35 protein (Q^22^-P^344^) could bind hemin and that HBP35 was suggested to possess a putative heme binding sequence (Y^50^CPGGK^55^). To determine the hemin binding region of HBP35, we constructed and purified rHBP35 (Q^22^-P^344^), rHBP35 (Q^22^-P^344 ^with C^48^S and C^51^S) and truncated rHBP35 (M^135^-P^344^) proteins with N-terminal histidine-tags using a histidine-tag purification system and carried out hemin binding assays using a hemoprotein peroxidase assay. As shown in Figure 4B, all of the rHBP35 (Q^22^-P^344^), rHBP35 (Q^22^-P^344 ^with C^48^S and C^51^S) and truncated rHBP35 (M^135^-P^344^) proteins were found to have hemin binding ability, implying that the hemin binding site is located in M^135^-P^344 ^of HBP35 protein.

### Effect of hemin depletion on growth of the hbp35 mutant

**Figure 5:**
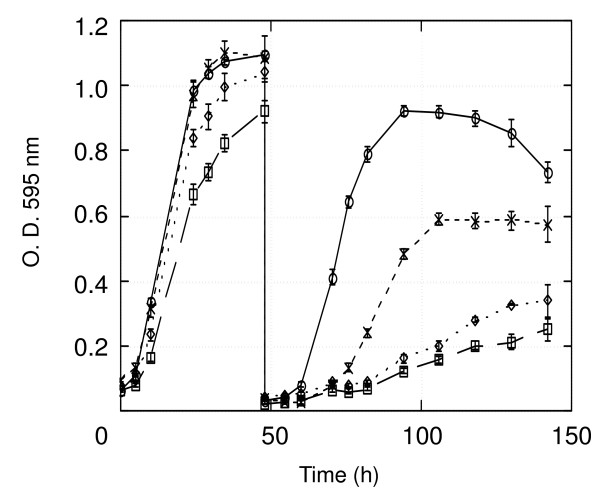
**Growth in hemin-containing BHI broth (0-48 h) and hemin-free BHI broth (after 48 h)**. Circle, 33277; square, KDP164 (*hbp35 *insertion mutant); diamond, KDP166 (*hbp35 *deletion mutant); X, KDP171 (*hbp35*^+ ^complemented strain derived from KDP166).

Since HBP35 protein is a hemin-binding protein, we determined the contribution of HBP35 proteins to acquisition or intracellular storage of heme. The *hbp35 *insertion mutant, the full length deletion mutant, the complemented strain which was constructed by replacing the intact *hbp35 *gene into the *hbp35 *full length deletion mutant, and the wild-type strain were hemin-starved after being grown in enriched BHI broth containing hemin (Figure 5). Hemin starvation resulted in retardation of the growth of the *hbp35 *mutants compared to that of the wild type, whereas the complemented strain partially recovered the growth retardation of the *hbp35 *deletion mutant under the hemin-depleted condition. Even under the hemin replete condition, the *hbp35 *mutants grew more slowly than the wild type, suggesting that HBP35 plays a role in hemin utilization in a sufficient hemin concentration (5 μg/ml).

### Thioredoxin activity of rHBP35 proteins

**Figure 6:**
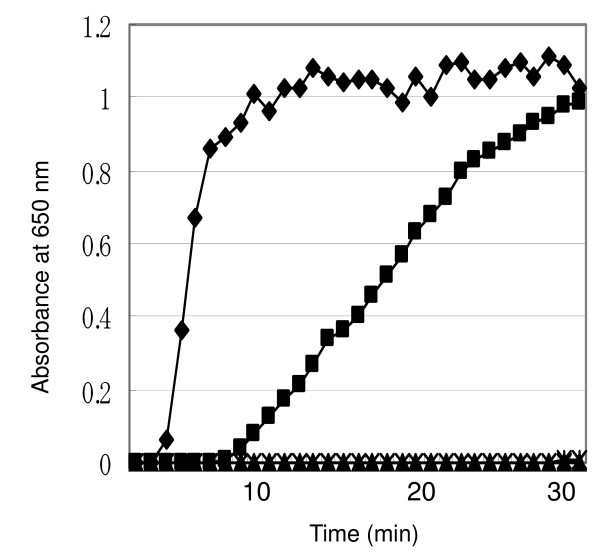
**Thioredoxin-catalyzed reduction of insulin by DTT**. Increase in turbidity at 650 nm was plotted against reaction time. Closed diamond, rHBP35(Q^22^-P^344^) plus DTT; closed square, *E. coli *thioredoxin plus DTT; closed triangle, rHBP35(Q^22^-P^344 ^with C^48^S C^51^S) plus DTT; X, rHBP35(Q^22^-P^344^) without DTT.

Shiroza *et al*. [12] have shown that an *hbp35 *gene-containing plasmid complemented the defects in motility and alkaline phosphatase activity of an *E. coli dsbA *mutant. This finding indicates that HBP35 is exported to the periplasm in a *dsbA *mutant and plays a role in the disulfide bond formation [13]. The HBP35 protein has a thioredoxin motif in the N-terminal region. We performed an insulin reduction assay to determine whether HBP35 has thioredoxin activity. Reduction of disulfide bonds of insulin by thioredoxin activity generates free A and B chains of insulin, and the resulting B chain is precipitated, which can be measured by the increase in turbidity [14]. The reducing activity of rHBP35 (Q^22^-P^344^) was higher than that of *E. coli *thioredoxin, whereas no activity was detected in rHBP35 (Q^22^-P^344 ^with C^48^S and C^51^S), indicating that HBP35 protein exhibits thioredoxin activity and that the two cysteine residues (C^48 ^and C^51^) are crucial for this activity (Figure 6).

### Diffuse bands of 50-90 kDa proteins are associated with anionic polysaccharide

**Figure 7:**
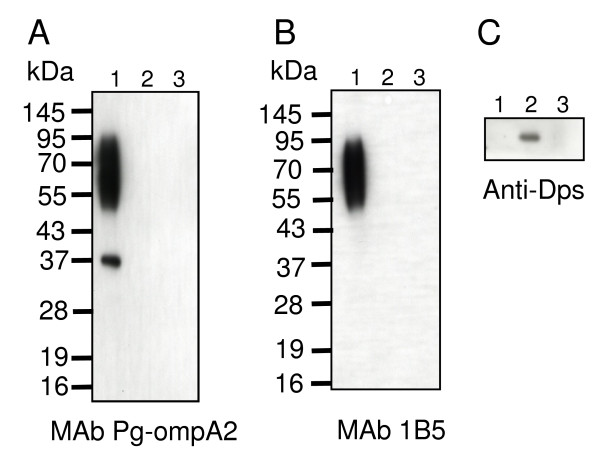
**Posttranslational glycosylation of HBP35 in *P. gingivalis *KDP136 (gingipain-null mutant)**. Immunoprecipitates with anti-HBP35 antibody (lane 1), with anti-Dps antibody (lane 2), and without an antibody (lane 3) were loaded on SDS-10% polyacrylamide gel and immunoblot analysis was performed with MAb Pg-ompA2 (A), MAb 1B5 (B), and anti-Dps antibody (C).

Nguyen *et al*. [11] revealed glycosylation of RgpB by immunoblot analysis with a monoclonal antibody (MAb 1B5) that recognizes the anionic polysaccharide of A-LPS [10,15]. To determine whether HBP35 is glycosylated, we carried out an immunoprecipitation experiment. Immunoprecipitates from the protein extracts of KDP136 (gingipain-null mutant) with an anti-HBP35 rabbit polyclonal antibody contained the 40-kDa protein and diffuse proteins of 50-90 kDa, which were revealed by immunoblot analysis with an anti-HBP35 mouse monoclonal antibody (MAb Pg-ompA2) [16]. The diffuse proteins of 50-90 kDa immunoreacted with MAb 1B5, indicating that HBP35 is associated with anionic polysaccharide on the cell surface (Figure 7). It is likely that the diffuse bands are HBP35 proteins binding to anionic polysaccharides with different numbers of repeating units.

### No diffuse bands were detected when the last five C-terminal residues of HBP35 were deleted

**Figure 8:**
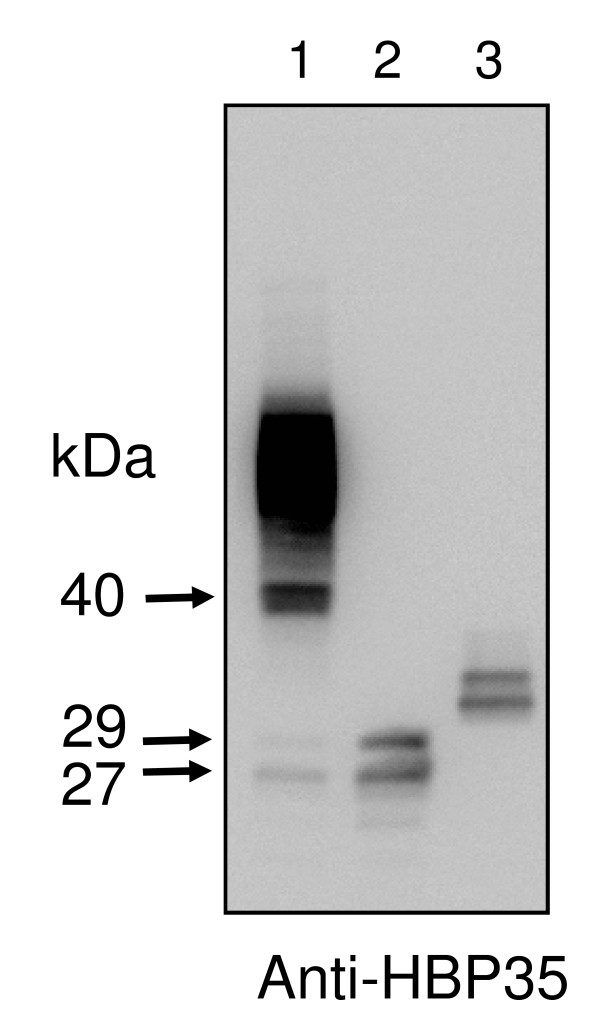
**Immunoblot analysis of cell extracts of various *P. gingivalis *strains with anti-HBP35 antibody**. Lane 1, 33277; lane 2, KDP164 (*hbp35 *insertion mutant); lane 3, KDP167 (*hbp35 *deletion of K^340^-P^344 ^mutant).

As HBP35 contains the C-terminal domain motif that may play a role in export and cell surface attachment, and that the presence of diffuse bands of 50-90 kDa proteins is similar to that of RgpB [8-11], it is possible that HBP35 is transported to the outer membrane and anchored to the cell surface by the same transport system as RgpB. Nguyen *et al*. [11] reported that the last five C-terminal residues (KVIVK) of RgpB play a significant role in the post-translational modification/proteolytic processing and exportation of proteins to the outer membrane. To determine whether the last five C-terminal residues (K^340^VLVP^344^) of HBP35 play a role similar to that of RgpB, we constructed an *hbp35 *deletion of K^340^-P^344 ^mutant and found that the mutant showed no diffuse bands but only 33-and 31-kDa proteins, which may have been generated by degradation of HBP35 protein accumulating in the cell (Figure 8). The result suggests that the last five C-terminal residues have an important role in the transport of HBP35 protein to the cell surface.

## Discussion

As *P. gingivalis *requires heme as the source of iron and protoporphyrin IX, a heme binding and transport system is essential for the microorganism to survive. Recently, several TonB-linked outer membrane receptors for heme utilization, including HmuR, Tlr, IhtA and HemR, have been reported [4]. The ability to store heme in bacterial cells appears to provide a nutritional advantage for survival of the bacterium in the iron-limited environment of a healthy gingival crevice [17]. In fact, heme can bind the *P. gingivalis *cell surface and may then be transported into the cell by an energy-dependent process [18]. Shibata *et al*. [7] found that purified rHBP35 protein (Q^22^-P^344^) could bind hemin but not hemoglobin or lactoferrin. HBP35 was suggested to possess a putative heme binding sequence (Y^50^CPGGK^55^), however, we found in this study that hemin could bind the mutant rHBP35 (Q^22^-P^344 ^with C^48^S and C^51^S) and the truncated rHBP35 (M^135^-P^344^) (Figure 4B), indicating that the hemin binding site is located between M^135 ^and P^344^. The *hbp35 *mutants grew more slowly than the wild type in hemin-depleted conditions and even in the condition with a sufficient hemin concentration (5 μg/ml), indicating that HBP35 protein plays a role in hemin utilization in various hemin levels.

The truncated HBP35 proteins of 27-and 29-kDa, which were derived from a 3'-portion of the *hbp35 *gene, were mainly located in the cytoplasm/periplasm fraction. This finding together with the fact that there is no signal peptide region in the two proteins suggests that these proteins are located in the cytoplasm and contribute to the intracellular storage of heme as does bacterioferritin (Figure 6). Similar protein expression has been found in *Neisseria meningitidis*: two forms of PilB protein are produced from the *pilB *gene. One is secreted to the outer membrane as a whole polypeptide with methionine sulfoxide reductase (Msr) A and B activities, and the other is a truncated form lacking an N-terminal thioredoxin domain, which is generated from an internal AUG initiation codon and is located in the cytoplasm [19].

Hiratsuka *et al*. [20] have previously reported that HBP35 shows no significant similarity with any other known proteins. As the truncated rHBP35 (M^135^-P^344^) protein has hemin binding activity, H^204^-H^206^, H^252^-H^253^, and H^261 ^within the truncated protein may interact with heme, in a similar fashion to the myoglobin and hemoglobin heme pockets in which two histidines hold heme through interaction with the central iron atom [21].

Recently, Dashper *et al*. [22] reported that expression of the *hbp35 *gene in strain W50 was not induced under a hemin-limited condition. We also observed that expression of the *hbp35 *gene in 33277 was not induced under hemin-depleted conditions (data not shown). Although HmuR, which is one of the hemin receptors, has been found to be regulated by one transcriptional activator [23], it seems unlikely that expression of the *hbp35 *gene is regulated by a specific transcriptional activator under hemin-depleted conditions.

Physiological roles of thioredoxins (Trxs) in *P. gingivalis *have not been established. In general, the intracellular environment is maintained in a reduced condition because of the presence of small proteins with redox-active cysteine residues, including Trxs, glutaredoxins (Grxs), monocysteine tripeptide glutathione (GSH) and other low-molecular-weight thiols [24,25]. In this regard, analysis of the *P. gingivalis *33277 and W83 genome sequences revealed the presence of thioredoxin reductase (TrxB; PGN1232 in 33277, PG1134 in W83), thioredoxin homologue (PGN0033 in 33277, PG0034 in W83), and 5 thioredoxin family proteins (PGN0373, PGN0488, PGN0659 (HBP35), PGN1181, and PGN1988 in 33277, PG0275, PG0616 (HBP35), PG1084, PG1638, and PG2042 in W83), and the absence of Grx, γ-glutamyl-_L_-cysteine-synthase and GSH synthetase. Recently, it has been shown that *Bacteroides fragilis*, which is phylogenetically close to *P. gingivalis*, possesses the TrxB/Trx system as the only reductive system for oxidative stress [26]. We previously showed that the thioredoxin protein (PGN0033) was increased when cells were exposed to atmospheric oxygen [27]. Although physiological roles of the thioredoxin domain of HBP35 protein are unknown at present, the diffuse bands of 50-90 kDa proteins, which contain the thioredoxin domain and are located on the outer membrane, may contribute to the maintenance of the redox status of the cell surface. However, we have not obtained a positive result indicating that HBP35 protein plays a role in protection against oxidative stresses so far.

Amino acid sequences in the RgpB that are necessary for transport of the protein to the outer membrane have been reported [8,11]. When recombinant truncated RgpB lacking its C-terminal 72 residues was produced in *P. gingivalis*, there was an altered distribution of the protein in the culture supernatant and periplasm. Seers *et al*. [8] reported the importance of the C-terminal domain of RgpB for attachment to the outer membrane and suggested that the domain is involved in a coordinated process of export and attachment to the cell surface. Nguyen *et al*. [11] found that the last five C-terminal residues of RgpB are conserved in a number of proteins of not only *P. gingivalis *but also other periodontal pathogens such as *Prevotella intermedia *and *Tannerella forsythia *and that they have an important role in mediating correct folding of the nascent protein, which is then transported across the periplasm to be fully glycosylated during its translocation across or on the outer membrane for anchorage to the outer leaflet of the outer membrane. The last five C-terminal residues of HBP35 (KVLVP) contain a stretch of polar-hydrophobic residues as well as those of RgpB (KVIVK). We found in this study that the diffuse bands of 50-90 kDa proteins, which were the main products of the *hbp35 *gene in the wild type, disappeared in the mutant strain lacking the last five C-terminal residues of HBP35, suggesting that, like RgpB, the C-terminal region of HBP35 plays an important role in transport of HBP35 to the outer membrane and anchorage to the membrane. Very recently, we found a novel protein secretion system (Por secretion system) in bacteria such as *P. gingivalis *belonging to phylum *Bacteroidetes *and suggested that the secretion system uses the C-terminal domain as a transportation signal [28]. HBP35 may therefore be transported to the cell surface via this secretion system.

The diffuse HBP35 protein bands of 50-90 kDa were immunoreactive with APS-recognizing MAb 1B5, indicating that a part of HBP35 protein is glycosylated, which is coordinated with the process of export. Rangarajan *et al*. [15] have recently shown that the anionic polysaccharide is associated with lipid A and they therefore renamed it LPS with APS repeating unit (A-LPS). HBP35 therefore as well as RgpB may be glycosylated on the cell surface by attachment to A-LPS.

## Conclusion

We found that the *hbp35 *gene produced a 1.1-kb transcript and several translational products; (i) a 40-kDa HBP35, which was derived from the whole *hbp35 *gene, was mainly located in the inner membrane, (ii) 29-and 27-kDa HBP35 proteins were N-terminal-truncated products lacking the signal peptide sequence and the thioredoxin domain and were mainly located in the cytoplasm, and (iii) diffuse HBP35 bands of 50-90 kDa proteins were glycosylated and located on the outer membrane. Analysis of these HBP35 proteins revealed that they played a significant role in heme acquisition. The last five C-terminal residues of HBP35 were crucial for the secretion to the outer membrane.

## Methods

### Bacterial strains and plasmids

All bacterial strains and plasmids used in this study are listed in Additional file 5.

### Media and conditions for bacterial growth

*P. gingivalis *strains were grown anaerobically (80% N_2_, 10% CO_2_, 10% H_2_) in enriched brain-heart infusion (BHI) broth (Becton Dickinson) or on enriched Trypto-soya (TS) agar plates (Nissui) supplemented with 5 μg/ml hemin (Sigma) and 0.5 μg/ml menadione (Sigma). Luria-Bertani (LB) broth and LB agar plates were used for growth of *E. coli *strains. Antibiotics were used at the following concentrations: ampicillin (Ap; 100 μg/ml for *E. coli*, 10 μg/ml for *P. gingivalis*), erythromycin (Em; 10 μg/ml for *P. gingivalis*), tetracycline (Tet; 0.7 μg/ml for *P. gingivalis*), kanamycin (Km; 50 μg/ml for *E. coli*).

### Chemicals

Proteinase inhibitors Nα-p-tosyl-_L_-lysine chloromethyl ketone (TLCK) and iodoacetamide were purchased from Wako, and leupeptin was obtained from Peptide Institute.

### Construction of P. gingivalis mutant strains

*P. gingivalis *W83 and 33277 genome sequence data were obtained from [GenBank: AE015924] and [GenBank: AP009380], respectively. The DNA primers used in this study are shown in Additional file 6. *P. gingivalis hbp35 *insertion mutant was constructed as follows. A DNA fragment corresponding to a region (0.80 kb) containing the C-terminal lower portion of PG0615 and the N-terminal upper portion of the PG0616 gene was generated by PCR using *P. gingivalis *W83 chromosomal DNA as the template with a forward primer, MS1, containing a *Kpn*I site (underlined) and a backward primer, MS2, containing an *Eco*RI site (underlined). The resulting fragment was cloned into the pGEM-T Easy vector (Promega) to yield pKD732. A DNA fragment corresponding to a region (0.70 kb) containing the C-terminal portion of the PG0616 gene was generated by PCR using *P. gingivalis *W83 chromosomal DNA as the template with a forward primer, MS3, containing a *Bgl*II site (underlined) and a backward primer, MS4, containing a *Not*I site (underlined). The resulting fragment was cloned into the pGEM-T Easy vector to yield pKD733. The *Bgl*II-*Not*I region of pKD733 containing the 0.70-kb fragment was swapped with both equivalent sites of pKD704 [29], resulting in pKD734. The *Kpn*I-*EcoR*I region of pKD732 containing the 0.80-kb fragment was swapped with both equivalent sites of pKD734, resulting in pKD735. Proper orientation of the pKD735 gene was confirmed by DNA sequence analysis. The pKD735 plasmid DNA was linearlized by *Not*I and introduced into *P. gingivalis *33277 by electroporation [29]. The cells were spread on TS agar containing 10 μg/ml Em and incubated anaerobically for 7 days. Proper sequence replacement of the Em-resistant transformants (KDP164 [insertion mutant]) was verified by Southern and Western blot analyses. *P. gingivalis hbp35 *whole gene deletion mutant from 33277 was constructed as follows. A DNA fragment corresponding to a region (0.49 kb) within the PG0615 gene and upstream region of PG0616 gene was generated by PCR using pMD125 [30] as the template with a forward primer, MS5, containing an *Sph*I site (underlined) and a backward primer, MS6, containing a *Bam*HI site (underlined). The resulting fragment was cloned into the pGEM-T Easy Vector to yield pKD737. A DNA fragment corresponding to a region (0.47 kb) located between the PG0617 gene and PG0618 gene upper region was obtained by PCR with a forward primer, MS7, containing a *Pst*I site (underlined) and a backward primer, MS8, containing an *Sac*I site (underlined). The resulting fragment was cloned into pCR4 (Invitrogen) to yield pKD738. The *Sph*I-*Bam*HI region of pKD737 containing the 0.49-kb fragment was inserted into the same sites of pAL30 [22] which contains the *ermF *gene in the pGEM-T Easy Vector and was located at the upper region of the *ermF *DNA block (1.2 kb), resulting in pKD739. The *Pst*I-*Sac*I site of pKD738 was inserted into the same sites of pKD739 that was located at the lower region of the *ermF *DNA block, resulting in pKD740. The pKD740 plasmid was linearlized by *Sac*I and introduced into *P. gingivalis *33277 by electroporation. Proper sequence replacement of the resulting Em-resistant transformant (KDP166 [deletion mutant]) was verified by PCR analysis.

### Plasmid construction for an hbp35 deletion (K^340^-P^344^) mutant

To create an *hbp35 *mutant lacking the last five amino acid residues (K^340^-P^344^), a DNA fragment corresponding to a region (1.5 kb) containing the C-terminal lower portion of PG0615 and PG0616 lacking K^340^-P^344 ^was generated by PCR using pMD125 as the template with a forward primer, MS9, containing a *Kpn*I site (underlined) and a backward primer, MS10, containing a *Bam*HI site (underlined). The resulting fragment was cloned into the pCR4 vector to yield pKD741. A DNA fragment corresponding to a region (0.47 kb) containing the PG0617 gene and PG0618 gene upper region was generated by PCR using pMD125 as the template with a forward primer, MS11, containing a *Bam*HI site (underlined) and a backward primer, MS12, containing a *Not*I site (underlined). The resulting fragment was cloned into the pGEM-T Easy Vector to yield pKD742. The *Bam*HI-*Not*I site of pKD742 was inserted into the same sites of pKD741 to yield pKD743. To create a *Bgl*II site located 8 bp upstream of PG0617 in pKD743, the two-stage PCR-based overlap extension method [31] was applied. MS9 and MS12, containing a *Not*I site (underlined), were used as external primers, and MS13, containing a *Bgl*II site (underlined), and MS14, containing a *Bgl*II site (underlined), were used as internal primers. Briefly, the amplified PCR fragments with MS9 and MS14 or with MS13 and MS12 were purified and further amplified with MS9 and MS12 primers by using both fragments as the template and was cloned into the pBluescript SK-, yielding pKD744. The *ermF-ermAM *DNA block (2.1 kb) from pKD399 [29] was inserted into the *Bgl*II site of pKD744 that was located at the junction of the 1.5-kb *hbp35 *gene-containing fragment and the 0.47-kb *hbp35 *downstream fragment to yield pKD745. The pKD745 plasmid was linearlized by *Not*I and introduced into *P. gingivalis *33277 by electroporation. Proper sequence replacement of the resulting Em-resistant transformant (KDP167) was verified by PCR analysis.

### Plasmid construction for an hbp35 gene complemented strain

To construct a strain where the *hbp35 *would be restored, the *Kpn*I-*Bgl*II site of pKD744 was swapped with the PCR fragment which was amplified by MS9 and a backward primer, MS14, containing a *Bgl*II site (underlined) using pMD125 as the template to yield pKD754, and then the *Bam*HI-*Bam*HI fragment containing the *cep*A DNA block by using CEPFOR and CEPREV primers from pCS22 was inserted into the *Bgl*II site of pKD754 to yield pKD755. The pKD755 plasmid was linearlized by *Not*I and introduced into KDP166 by electroporation. Proper sequence replacement of the resulting Ap-resistant transformant (KDP171) was verified by PCR and immunoblot analyses.

### Site-directed mutagenesis

To create *hbp35 *insertion mutants with M^115^A and/or M^135^A, site-directed mutagenesis was performed using a QuickChange II Site-Directed Mutagenesis kit (Stratagene, La Jolla, CA, USA). The *hbp35 *insertion mutant targeting vector containing M^115^A substitution (pKD746) was constructed with the oligonucleotide sense primer MS15, containing an M^115^A substitution (underlined), and antisense primer MS16, containing an M^115^A substitution (underlined), and the recombinant plasmid pKD735 as the template. The *hbp35 *insertion mutant targeting vectors containing M^135^A (pKD747) or M^115^A M^135^A substitutions (pKD748) were constructed with the oligonucleotide sense primer MS17, containing an M^135^A substitution (underlined), and antisense primer MS18, containing an M^135^A substitution (underlined), and the recombinant plasmid pKD735 and pKD746 as the template. To create *hbp35*[M^115^A], *hbp35*[M^135^A] or *hbp35*[M^115^A M^135^A] insertion mutants which had an insertion with the *ermF-ermAM *DNA cassette that was located just upstream of F^110^, pKD746, pKD747 and pKD748 were linearlized with *Not*I and introduced into *P. gingivalis *33277, giving KDP168, KDP169 and KDP170, respectively.

### Construction of expression plasmids

To create a recombinant HBP35 protein (A^1^-P^344^) with a C-terminal histidine-tag overexpression system, a 1.0-kb PCR fragment was amplified using forward primer MS19, containing an *Nco*I site (underlined) and backward primer MS20, containing an *Xho*I site (underlined), and then cloned into the pCR4 to yield pKD749. The *Eco*RI-*Xho*I sites of pKD749 were inserted into the same sites of pET21d(+), resulting in pKD750. To create a recombinant HBP35 protein (Q^22^-P^344^) with an N-terminal histidine-tag overexpression system, a 0.97-kb PCR fragments were amplified using forward primer MS21 and backward primer MS22 and then cloned into the pET30 Ek/LIC vector (Novagen), resulting in pKD751. Site-directed mutagenesis of the thioredoxin active site in HBP35 was performed using a QuickChange II Site-Directed Mutagenesis kit. A double amino acid substitution mutant (rHBP35 Q^22^-P^344 ^with C^48^S C^51^S) was created with the oligonucleotide sense primer MS23, containing C^48^S and C^51^S substitutions (underlined), and antisense primer MS24, containing C^48^S and C^51^S substitutions (underlined), using the recombinant plasmid pKD751 as the template to yield pKD752. Proper mutation was confirmed by DNA sequencing. To create a recombinant truncated HBP35 protein (M^135^-P^344^) with an N-terminal histidine-tag overexpression system, a 0.66-kb PCR fragments were amplified using forward primer MS25 and backward primer MS22, and then cloned into pET30Ek/LIC vector, resulting in pKD753.

### Expression and purification of P. gingivalis recombinant HBP35 proteins

*E. coli *BL21(DE3)pLysS harboring pKD750, pKD751, pKD752 or pKD753 was cultured in LB medium containing 100 μg/ml of Ap at 37°C to OD_600 _of 0.4-0.6, and then IPTG was added to the culture at 1 mM, followed by an additional 3-h incubation. The cells were harvested, suspended in buffer A (50 mM NaH_2_PO_4 _[pH 8.0], 500 mM NaCl, 10 mM imidazole) and then disrupted with a French Press. The mixture was centrifuged at 3,000 × g for 15 min to separate the inclusion body fraction (pellet) from the soluble fraction (supernatant). The supernatant was loaded onto a pre-equilibrated Ni^2+^-NTA agarose column (Invitrogen) of 2 ml in bed volume and incubated at 4°C for 30 min. The column was washed three times with buffer B (50 mM NaH_2_PO_4 _[pH 8.0], 500 mM NaCl, 20 mM imidazole) and the bound protein was eluted with 10 ml of elution buffer (50 mM NaH_2_PO_4 _[pH 8.0], 500 mM NaCl, 250 mM imidazole) as 1-ml fractions. The fractions were analyzed by SDS-PAGE. The pure fractions were pooled and then dialyzed against milliQ water and stored at -20°C until further use. N-terminal amino acid sequencing (Edman sequencing) of the purified rHBP35 protein with the C-terminal histidine-tag was carried out using the service facility in CSIRO (Melbourne, Australia).

### Gel electrophoresis and immunoblot analysis

SDS-PAGE was performed according to the method of Laemmli [32]. Protease inhibitors (leupeptin and TLCK) were added to Laemmli solubilizing buffer to avoid proteolysis by endogenous proteases. The gels were stained with 0.1% Coomassie Brilliant Blue R-250 (CBB). For immunoblotting, proteins on SDS-PAGE gels were electrophoretically transferred onto polyvinylidene fluoride (PVDF) membranes (Immobilon P; Millipore) as described previously [33]. The blotted membranes were detected with an anti-HBP35 polyclonal antibody [6].

### Preparation of P. gingivalis subcellular fractions

*P. gingivalis *cells were harvested from 400 ml of fully-grown culture by centrifugation at 10,000 × g for 30 min at 4°C, washed twice with 10 mM HEPES-NaOH (pH 7.4) containing 0.15 M NaCl, and resuspended in 20 ml of HEPES containing 0.1 mM TLCK, 0.1 mM leupeptin and 0.2 mM PMSF. The cells were disrupted with a French Press by three passes at 100 MPa in the presence of 25 μg/ml each of RNase and DNase. Unbroken cells were removed by centrifugation at 1,000 × g for 10 min and the supernatant was subjected to ultracentrifugation at 100,000 × g for 60 min. The precipitates were treated with 1% Triton-X100 in HEPES containing 20 mM MgCl_2 _for 30 min at 20°C. The inner and outer membrane fractions were recovered as a supernatant and a pellet, respectively, by ultracentrifugation at 100,000 × g for 60 min at 4°C [34].

### In-gel digestion of proteins and Peptide Mass Fingerprinting

To identify the 27-kDa protein, *P. gingivalis *KDP161 cells were harvested, and the cell pellets were dissolved with RIPA buffer (150 mM NaCl, 1% Nonidet P-40, 0.5% deoxycholate, 0.1% SDS and 50 mM Tris-HCl, pH 8.0) and then immunoprecipitated by EZview red protein A affinity gel (Sigma) with anti-HBP35 polyclonal antibody, followed by SDS-PAGE analysis with CBB staining and immunoblot analysis. Protein bands from the SDS-PAGE gel were excised and subjected to in-gel tryptic digestion as described previously [8,9]. Gel pieces were washed in 50 mM NH_4_HCO_3_-ethanol (1:1, vol/vol), reduced, alkylated with dithiothreitol and iodoacetamide, respectively, and digested with sequencing-grade modified trypsin (10 ng/μl) (Promega) overnight at 37°C. Each digest (0.5 μl) was then analyzed by mass spectrometry using an Ultraflex TOF/TOF instrument (Bruker Daltonics, Bremen, Germany) in positive-ion and reflectron mode. A saturated solution of α-cyano-4-hydroxycinnamic acid was prepared in 97:3 (vol/vol) acetone-0.1% aqueous trifluoroacetic acid (TFA). A thin layer of matrix was prepared by pipetting and immediately transferring 2 μl of this solution onto 600-μm anchors of an AnchorChip target plate (Bruker Daltonics). The tryptic digest sample (0.5 μl) was then deposited onto the thin layers with 2.5 μl of 0.1% (vol/vol) TFA for 1 min. Mass spectra were calibrated by external calibration using a standard peptide mix (Bruker Daltonics). Proteins were identified by PMF against the *P. gingivalis *database (available at The Institute for Genomic Research [TIGR] website [http://www.tigr.org]) using an in-house Mascot search engine (Matrix Science Ltd., London, United Kingdom) and BioTools 2.2 software (Bruker Daltonics) and by comparison with tryptic peptide mass lists generated by using General Protein Mass Analysis for Windows software (Lighthouse Data, Odense, Denmark).

### Northern blot analysis

Total RNA extraction and Northern blot analysis of mRNA were carried out as described previously [35] with some modifications. The 0.96-kb DNA fragment coding for Q^22 ^to P^344 ^of HBP35 and the 0.80-kb DNA fragment coding for M^1 ^to S^266 ^of ErmF were obtained by PCR that were used as the radiolabelled *hbp35 *and *ermF *probes, respectively. To label the DNA probes, [α-^32^P]dCTP and the ready-to-go DNA labeling beads kit (GE Healthcare) was used. The radiolabelled products were analyzed with a fluoro-image analyzer FLA-5100 (Fujifilm).

### Hemin binding assay

Hemin binding to rHBP35 proteins was assayed using the catalytic property of hemoprotein, which has peroxidase activity in the presence of H_2_O_2_, by the method of Shibata *et al*. [7] with some modifications. Ten microliters of protein solution (2 μg) was treated with 1.5 μl of 1.25 mM hemin for 2 h at room temperature. After SDS-PAGE, the gel was washed twice for 30 min in TBS buffer (10 mM Tris-HCl, pH 7.5, 0.9% NaCl) and then exposed to a reaction buffer (1 mg of 4-methoxy-1-naphthol, 20 μl H_2_O_2 _in 50 ml TBS buffer) for 30 min at room temperature.

### Hemin starvation

To determine the ability for growth under hemin starvation conditions, bacterial strains to be tested were first grown in the presence of hemin for 48 h and then deprived of hemin. The overnight cultures were prepared by growing the strains in hemin-containing enriched BHI broth overnight. In the case of first grown in hemin-containing BHI broth for 48 h, the overnight cultures were diluted 50-fold with hemin-containing BHI broth. Then the first grown bacterial cultures to be tested were diluted 50-fold with hemin-free BHI broth. The cell density of the culture was measured at OD_595_.

### Insulin reduction assay

A fresh solution of 1 mg/ml insulin was prepared in 100 mM potassium phosphate, 2 mM EDTA, pH 7.0. The assay mixture contained a total volume of 800 μl of 100 mM potassium phosphate, 2 mM EDTA, pH 7.0, 0.13 mM insulin, 1 mM DTT, and 1 μM of freshly purified recombinant histidine-tagged HBP35 protein in the standard insulin disulfide reduction assay [14]. The increase in turbidity due to formation of the insoluble insulin B chain was measured at OD_650 _and 30°C. One micromolar fresh *E. coli *thioredoxin 1 (Sigma) was used as a positive control.

### Immunoprecipitation experiment

The harvested *P. gingivalis *KDP136 (gingipain-null mutant) cells [36] were dissolved with RIPA buffer (150 mM NaCl, 1% Nonidet P-40, 0.5% deoxycholate, 0.1% SDS and 50 mM Tris-HCl, pH 8.0) under absence of protease inhibitors and immunoprecipitated by protein G agarose beads (GE Healthcare) with 5 μg of anti-HBP35 polyclonal antibody or 5 μg of anti-Dps polyclonal antibody, or without an antibody. Each resulting precipitate was dissolved with the same volume of the sample buffer and loaded on an SDS-10% polyacrylamide gel. Immunoblot analysis was performed with MAb 1B5 [10], MAb Pg-ompA2 [16] and anti-Dps antibody [37].

## Authors' contributions

MS, YA, ECR and KN designed the study. MS wrote the manuscript with BP, ECR and KN. MS, YS, TS, HY, BP, YYC, KS and MN performed the experiments in this study. Especially, MS participated in almost all of the study, HY measured gingipain activity, YYC performed MALDI-TOF mass spectrometric analysis, and MN performed hemagglutinating assay. All authors read and approved the final manuscript.

## Supplementary Material

Additional file 1 **Northern blot analysis of *hbp35 *mRNA**. Total RNAs were electrophoresed, blotted, hybridized with the *hbp35 *DNA probe (left) or the *ermF *DNA probe (right), and subjected to autoradiography (see Methods). Lane 1, 33277; lane 2, KDP164 (*hbp35 *insertion mutant); lane 3, KDP166 (*hbp35 *deletion mutant). Click here for file

Additional file 2 **Preparation of the anti-HBP35-immunoreactive 27-kDa protein for PMF analysis**. Immunoprecipitates of lysates of KDP164 (*hbp35 *insertion mutant) with anti-HBP35 antibody was analyzed by SDS-PAGE followed by staining with CBB (left) or immunoblot analysis with anti-HBP35 antibody (right). A 27-kDa protein band on the gel indicated was subjected to PMF analysis. Click here for file

Additional file 3 **Structures of the HBP35 protein and the *hbp35 *gene**. A. Domain organization of HBP35 protein. HBP35 contains a signal peptide region, a thioredoxin domain and a C-terminal domain. B. The *hbp35 *gene loci in various mutant strains. Mutated *hbp35 *genes of KDP164 (*hbp35 *insertion mutant), KDP168 (*hbp35 *[M115A] insertion mutant), KDP169 (*hbp35 *[M135A] insertion mutant) and KDP170 (*hbp35 *[M115A M135A] insertion mutant) were depicted. Click here for file

Additional file 4 **N-terminal amino acid sequencing of the recombinant 27-kDa protein produced in an *E. coli *expressing the *hbp35 *gene**. rHBP35 products, which were partially purified using a C-terminal histidine-tag, were analyzed by SDS-PAGE followed by staining with CBB (left) or immunoblot analysis with anti-HBP35 antibody (right). The N-terminal amino acid sequence of the recombinant 27-kDa protein was determined by Edman sequencing, resulting in M^135 ^as an N-terminal residue. Click here for file

Additional file 5 **Bacterial strains and plasmids used in this study**. Click here for file

Additional file 6 **Oligonucleotides used in this study**. Click here for file
