# Supplementary figures and images for: Characterization of hemin-binding protein 35 (HBP35) in Porphyromonas gingivalis: its cellular distribution, thioredoxin activity and role in heme utilization
Source: BMC Microbiol. 2010 May 25;10:152. doi: 10.1186/1471-2180-10-152 (PMC2907840; doi:10.1186/1471-2180-10-152)

## Slide 1
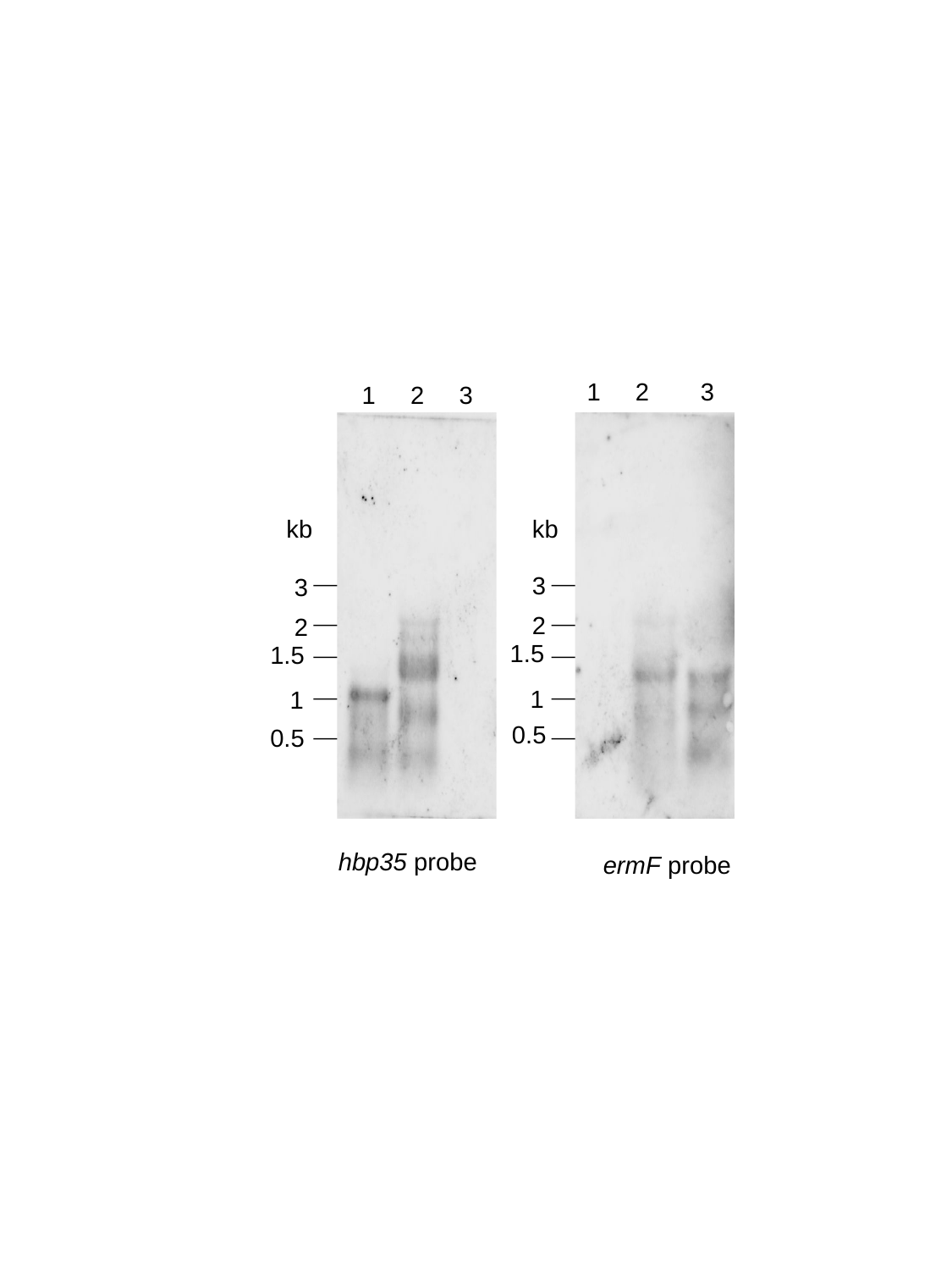

1 2 　 3
1 2 3
kb
kb
3
3
2
2
1.5
1.5
1
1
0.5
0.5
hbp35 probe
ermF probe

Supplement: Additional file 1 — Northern blot analysis of hbp35 mRNA. Total RNAs were electrophoresed, blotted, hybridized with the hbp35 DNA probe (left) or the ermF DNA probe (right), and subjected to autoradiography (see Methods). Lane 1, 33277; lane 2, KDP164 (hbp35 insertion mutant); lane 3, KDP166 (hbp35 deletion mutant). [file 1471-2180-10-152-S1.PPT]

## Slide 1
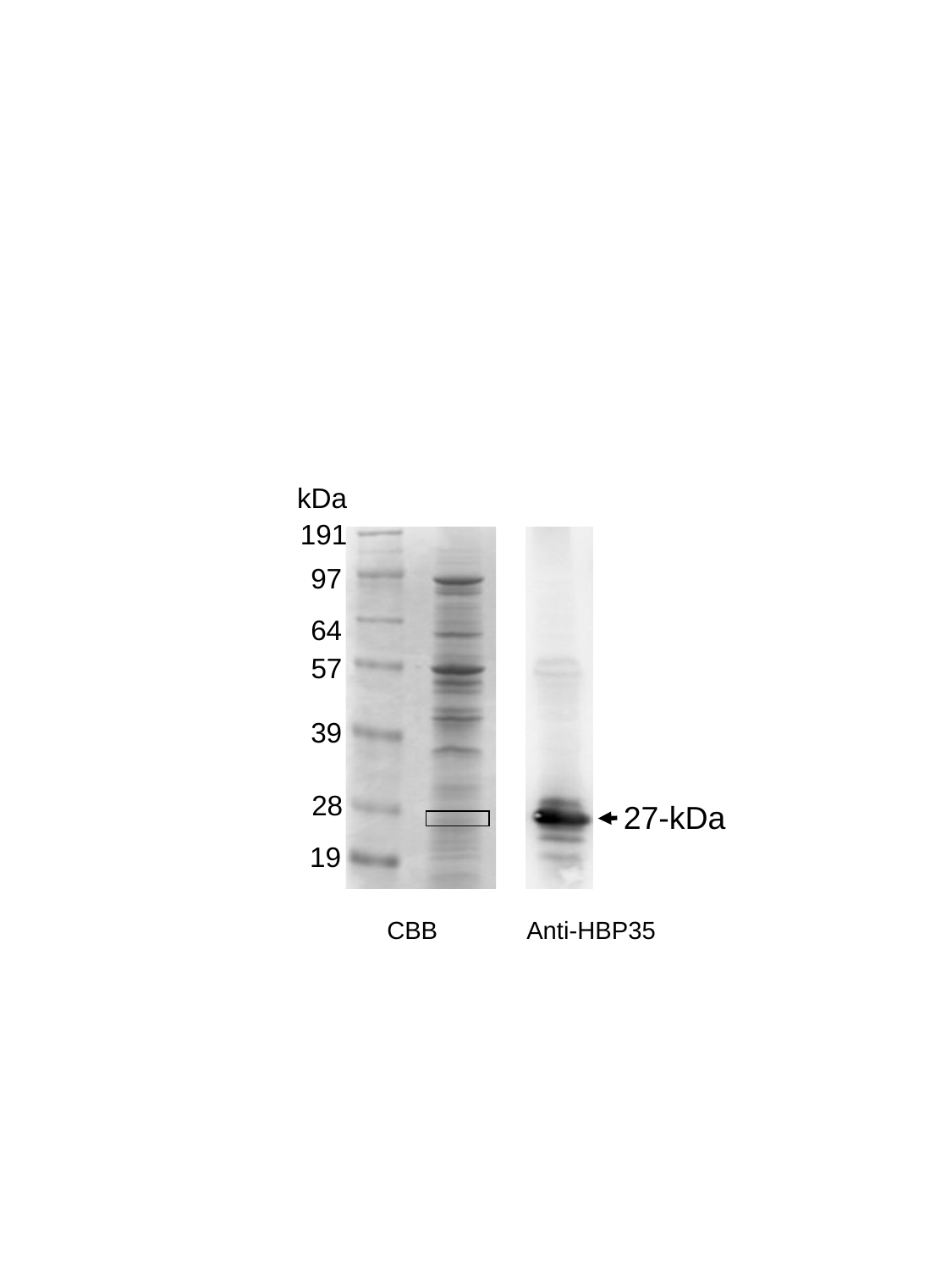

kDa
191
97
64
57
39
28
27-kDa
19
CBB
Anti-HBP35

Supplement: Additional file 2 — Preparation of the anti-HBP35-immunoreactive 27-kDa protein for PMF analysis. Immunoprecipitates of lysates of KDP164 (hbp35 insertion mutant) with anti-HBP35 antibody was analyzed by SDS-PAGE followed by staining with CBB (left) or immunoblot analysis with anti-HBP35 antibody (right). A 27-kDa protein band on the gel indicated was subjected to PMF analysis. [file 1471-2180-10-152-S2.PPT]

## Slide 1
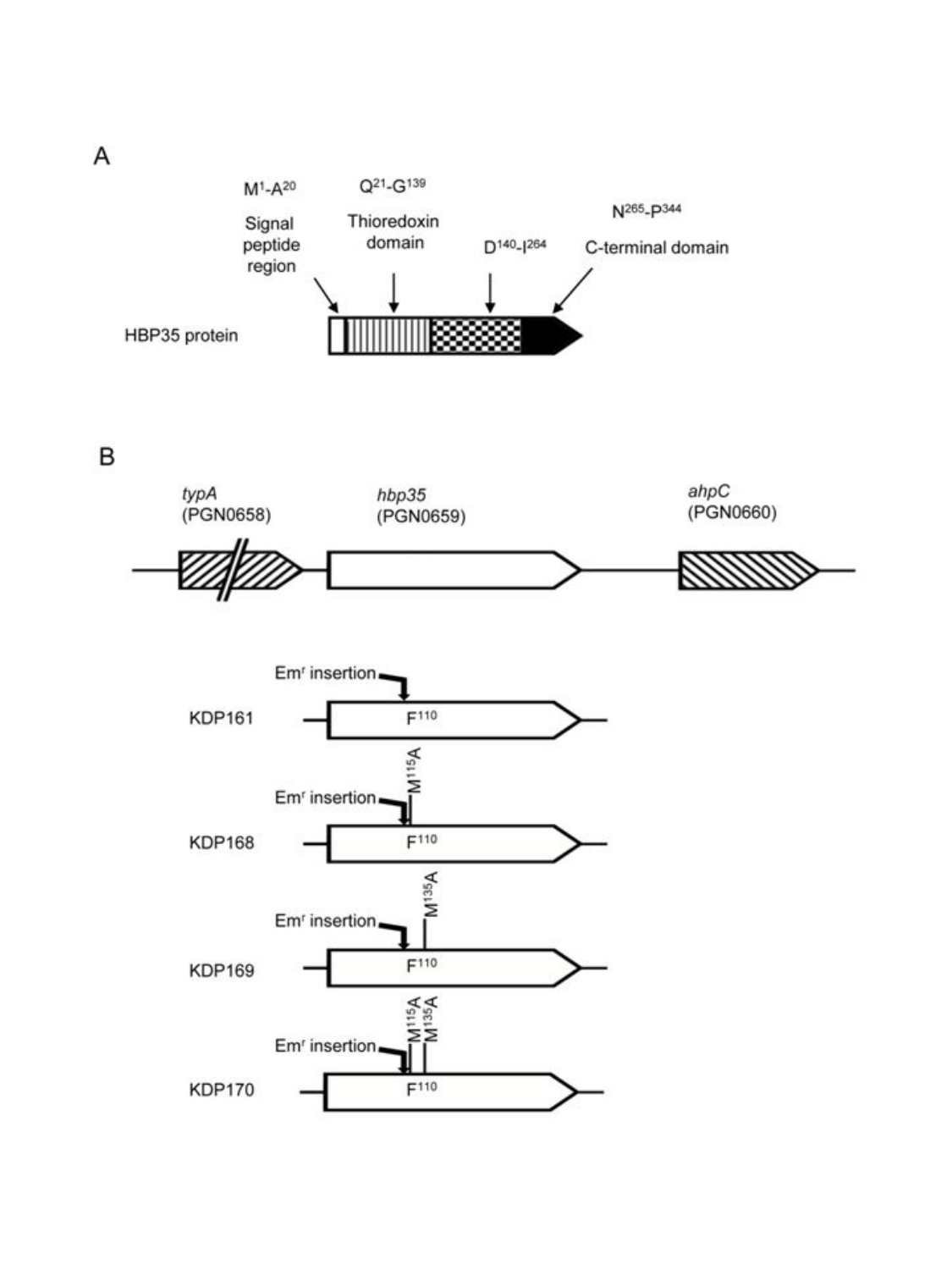

Supplement: Additional file 3 — Structures of the HBP35 protein and the hbp35 gene. A. Domain organization of HBP35 protein. HBP35 contains a signal peptide region, a thioredoxin domain and a C-terminal domain. B. The hbp35 gene loci in various mutant strains. Mutated hbp35 genes of KDP164 (hbp35 insertion mutant), KDP168 (hbp35 [M115A] insertion mutant), KDP169 (hbp35 [M135A] insertion mutant) and KDP170 (hbp35 [M115A M135A] insertion mutant) were depicted. [file 1471-2180-10-152-S3.PPT]

## Slide 1
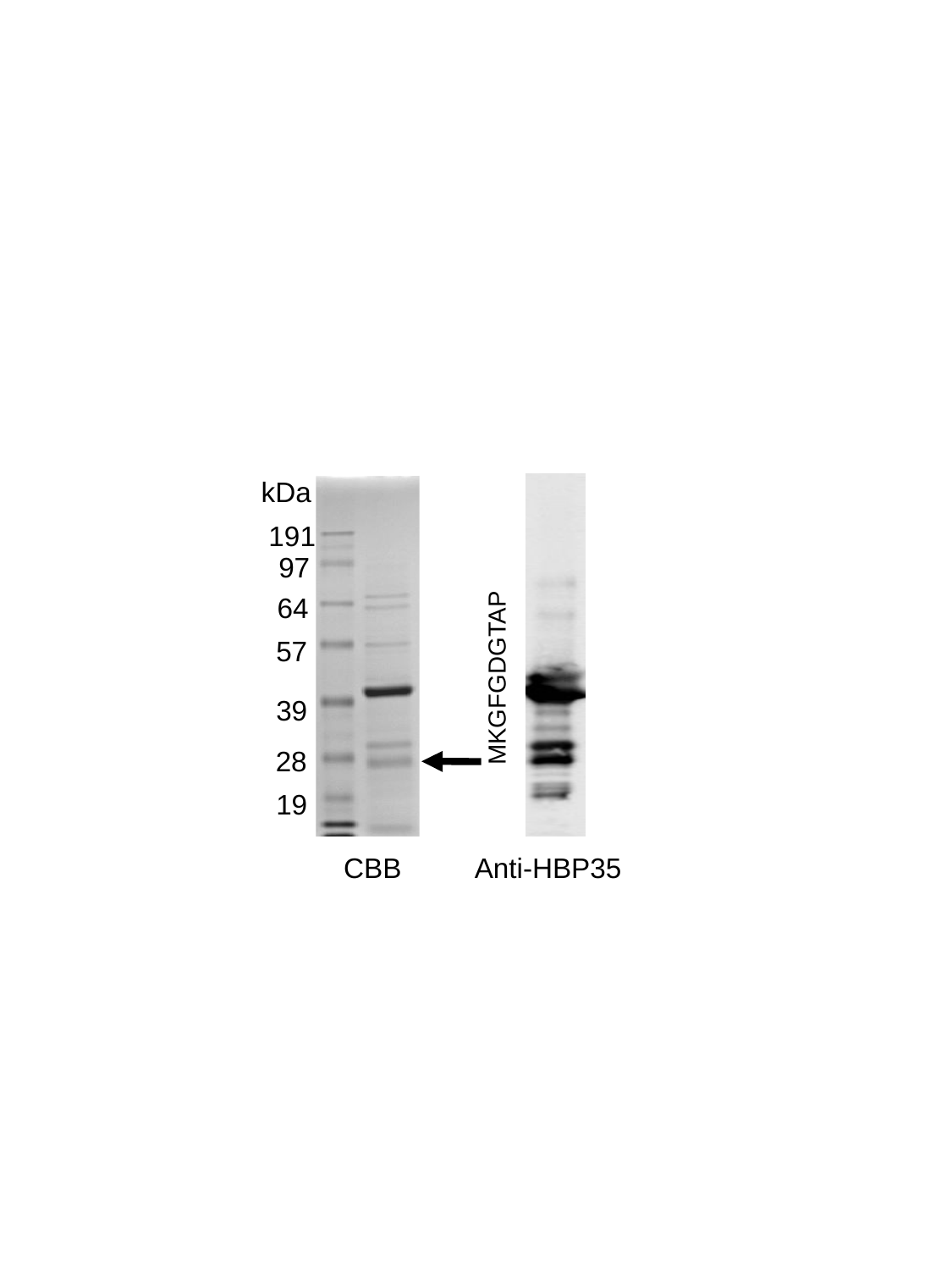

kDa
191
97
64
57
MKGFGDGTAP
39
28
19
CBB
Anti-HBP35

Supplement: Additional file 4 — N-terminal amino acid sequencing of the recombinant 27-kDa protein produced in an E. coli expressing the hbp35 gene. rHBP35 products, which were partially purified using a C-terminal histidine-tag, were analyzed by SDS-PAGE followed by staining with CBB (left) or immunoblot analysis with anti-HBP35 antibody (right). The N-terminal amino acid sequence of the recombinant 27-kDa protein was determined by Edman sequencing, resulting in M135 as an N-terminal residue. [file 1471-2180-10-152-S4.PPT]
