# Supplementary material for: Characterization of hemin-binding protein 35 (HBP35) in Porphyromonas gingivalis: its cellular distribution, thioredoxin activity and role in heme utilization
Source: BMC Microbiol. 2010 May 25;10:152. doi: 10.1186/1471-2180-10-152 (PMC2907840; doi:10.1186/1471-2180-10-152)
Supplement: Additional file 6 — Oligonucleotides used in this study. [file 1471-2180-10-152-S6.DOC]

Additional file 6. Oligonucleotides used in this study.

| Primer | Nucleotide sequences (5') |
| --- | --- |
| MS1 | GGGTACCCAGCGGCGCAGCAACGGGTCG |
| MS2 | GGGATTCCAAAAGTCGGAAAACCCGGAA |
| MS3 | AAGATCTTTCAGTTTGCCGTATGGAGAA |
| MS4 | GGCGGCCGCCTCA AGGAACCAAGACTTTAA |
| MS5 | GCATGCCAACCGTGGAAAGGGGAGATAGAGCGG |
| MS6 | GGATCCGTTCGTTTATCTTTTTTGTTAGTG |
| MS7 | CTGCAGTTTATTGAGCTAAGATTTAAACGA |
| MS8 | GAGCTCGCCATTGTGATAGGCATTGAGTTT |
| MS9 | GGTACCCAACCGTGGAAAGGGGAGATAGAG |
| MS10 | GGATCCTCAAAGGAAATGCATTACACCGTT |
| MS11 | GGATCCTTTATAGAGCTAAGATTTAAACGA |
| MS12 | GGCGGCCGCGCCATTGTGATA |
| MS13 | AAGATCTTCCATTCTCGGG |
| MS14 | AGATCTATGAGGAAAACGAAAACTGCGCCT |
| MS15 | CAGTTTGCCGTGCAGAGAAAAAAGG |
| MS16 | CTTTTTTCTCTGCACGGCAAACTG |
| MS17 | GCAATTAAAAATAAGATTGCAAAAGGTTTTGGTGATGG |
| MS18 | CCATCACCAAAACCTTTTGCAATCTTATTTTTAATTGC |
| MS19 | CCATGGCCATGAAAAGATTATTACTCTCTG |
| MS20 | CTCGAGAGGAACCAAGACTTTAAGGAAATA |
| MS21 | GACGACGACAAGATGCAAGAGTTGAAAACCTCTGCTGACATGAAA |
| MS22 | GAGGAGAAGCCCGGTTATCAAGGAACTAAGACTTTAAGGAAATG |
| MS23 | GTATTTACTGCCGAATGGTCCGGTTACTCTCCAGGTGGTAAAGAG |
| MS24 | CTCTTTACCACCTGGAGAGTAACCGGACCATTCGGCAGTAAATAC |
| MS25 | GACGACGACAAGATGAAAGGTTTTGGTGATGGTACA |
| CEPFOR | GGATCCGACGTCAAAAGAGTTAAGGAAAGTGAAGC |
| CEPREV | GGATCCGACGTCTTTCAAGTCACCGATAGTG |
